# Endobronchial actinomycosis

**DOI:** 10.1590/0037-8682-0315-2022

**Published:** 2022-10-24

**Authors:** Buğra Kerget, Ferhan Kerget, Sevilay Özmen

**Affiliations:** 1 Ataturk University School of Medicine, Department of Pulmonary Diseases, Yakutiye, Erzurum, Turkey.; 2 Health Sciences University Erzurum Regional Education and Research Hospital, Depertmant of Infection Diseases and Clinical Microbiology, Erzurum, Turkey.; 3 Ataturk University School of Medicine, Department of Pathology, Yakutiye, Erzurum, Turkey.

**FIGURE 1:**
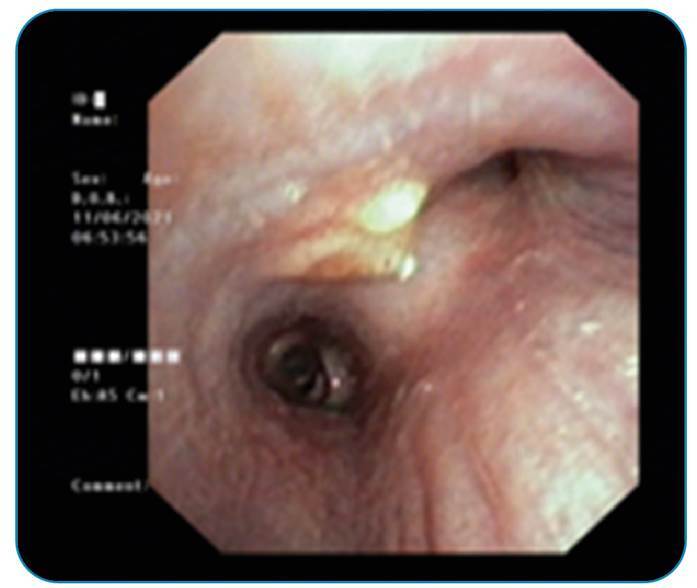
Nodular lesion from the mucosa is observed in the carina of the right lung upper lobe intermediary bronchus.

**FIGURE 2:**
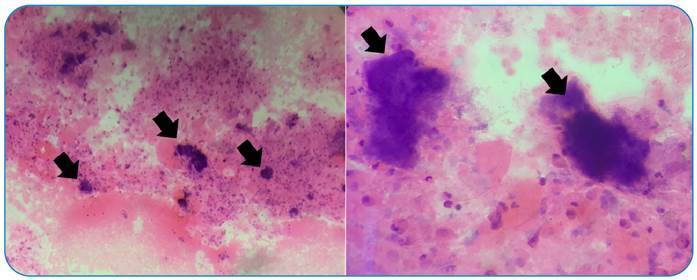
Nodular lesion is reported in accordance with actinomycosis (Hematoxylin-eosin x10, x100).

A 23-year-old woman was admitted to our outpatient clinic with an 8-week history of dry cough and shortness of breath that had developed with exertion. We observed no significant features in the patient's physical examination or laboratory tests. To evaluate mediastinal involvement, thoracic computed tomography was performed. It revealed a 73 x 69 mm cystic necrotic hypodense region in the right mediastinum, extending from lymph node stations two to four. In the paracardial region of the upper lobe of the right lung, there was a consolidated patch in the parenchyma that resembled a budded branch. Mediastinoscopy was scheduled to check for suspected malignancy, and biopsy material taken from the 2R localization was diagnosed as classic Hodgkin's lymphoma. Bronchoscopy was performed before treatment for the radiological finding of an infectious nature in the upper lobe. Bronchoscopy revealed a raised nodular lesion in the mucosa of the carina of the right lung upper lobe intermediary bronchus, from which a biopsy was extracted (Figure 1). However, the pathology report of the nodular lesion revealed actinomycosis (Figure 2). Before initiating treatment for hematologic malignancy, 6 × 4.000.000 units of crystallized penicillin were administered. Pulmonary actinomycosis remains a significant problem for clinicians due to the lack of specific symptoms and its similarity to other chronic sweeping chest diseases and malignancies^1^ ^,^ ^2^. Endobronchial actinomycosis is a rare condition, and histopathological evaluation invariably confirms its diagnosis. We aimed to contribute to the literature by presenting this condition, which could be treated with proper diagnosis and antibiotic therapy.
